# Transcriptome-wide association study reveals increased neuronal *FLT3* expression is associated with Tourette’s syndrome

**DOI:** 10.1038/s42003-022-03231-0

**Published:** 2022-03-30

**Authors:** Calwing Liao, Veikko Vuokila, Hélène Catoire, Fulya Akçimen, Jay P. Ross, Cynthia V. Bourassa, Patrick A. Dion, Inge A. Meijer, Guy A. Rouleau

**Affiliations:** 1 Department of Human Genetics, McGill University, Montréal, QC Canada; 2 Montreal Neurological Institute, McGill University, Montréal, QC Canada; 3 Department of Neurology and Neurosurgery, McGill University, Montréal, QC Canada; 4 Department of Neurosciences and CHU Sainte-Justine, University of Montréal, Montreal, QC Canada

**Keywords:** Neurological disorders, Neurodevelopmental disorders

## Abstract

Tourette’s Syndrome (TS) is a neurodevelopmental disorder that is characterized by motor and phonic tics. A recent TS genome-wide association study (GWAS) identified a genome-wide significant locus. However, determining the biological mechanism of GWAS signals remains difficult. To characterize effects of expression quantitative trait loci (eQTLs) in TS and understand biological underpinnings of the disease. Here, we conduct a TS transcriptome-wide association study (TWAS) consisting of 4819 cases and 9488 controls. We demonstrate that increased expression of *FLT3* in the dorsolateral prefrontal cortex (DLPFC) is associated with TS. We further show that there is global dysregulation of *FLT3* across several brain regions and probabilistic causal fine-mapping of the TWAS signal prioritizes *FLT3* with a posterior inclusion probability of 0.849. After, we proxy the expression with 100 lymphoblastoid cell lines, and demonstrate that TS cells has a 1.72 increased fold change compared to controls. A phenome-wide association study also points toward *FLT3* having links with immune-related pathways such as monocyte count. We further identify several splicing events in *MPHOSPH9*, *CSGALNACT2* and *FIP1L1* associated with TS, which are also implicated in immune function. This analysis of expression and splicing begins to explore the biology of TS GWAS signals.

## Abstract (web summary)

Calwing Liao and Veikko Vuokila et al. report a transcriptome-wide association study (TWAS) of Tourette’s Syndrome. They find increased expression of *FLT3* in the dorsolateral prefrontal cortex and the lymphoblastoid cell lines in patients with Tourette’s Syndrome, and report dysregulation of *FLT3* across several brain regions.

## Introduction

Tourette’s Syndrome (TS) is a neuropsychiatric disorder that is characterized by motor and phonic tics^1^. The onset of the disorder is typically between the age of 5 and 7 years. TS has been shown to have a large genetic component, in which the first-degree relatives of TS patients have a 10–100-fold higher rate of TS compared to the general population^2,3^. Past genetic studies of TS have identified several implicated genes such as *CELSR3*, a gene where recurrent do novo variants are found in probands^4^. Furthermore, a recent genome-wide association study (GWAS) identified a genome-wide significant hit on chromosome 13, rs2504235, which is within the *FLT3* (*Fms Related Tyrosine Kinase 3*) gene^5^. Although GWAS is a powerful method for identifying associated genetic loci, it is often difficult to interpret the biological effects of significant hits.

Recently, transcriptomic imputation was developed to allow for the integration of genetic and expression data from datasets such as the CommonMind (CMC) and Genotype-Tissue Expression (GTEx) consortia^6,7^. The derivation of panels involves a machine-learning approach to characterize the relationship between gene expression and genotypes, making tissue-specific predictive models. Transcriptomic imputation can leverage these reference imputation panels from these consortia and identifies the genetic correlation between imputed expression and GWAS data^6^. Ultimately, transcriptomic imputation allows for better characterization of GWAS data by prioritizing tissue-specific genes associated with disease^8^. Furthermore, imputation of aberrant splicing can be done through this method. Given that alternative splicing occurs frequently in brain tissue and in early development, identifying potential genes with aberrant splicing could be important for understanding the genetic etiology of brain disorders^9,10^. This methodology, known as a transcriptome-wide association study (TWAS), has already been used to prioritize genes in a variety of different traits, such as schizophrenia, depression, and ADHD^6,11–14^. For instance, for ADHD, a TWAS was done with adult brain tissue and found several genes including *ST3GAL3*^15^. A subsequent ADHD TWAS using fetal brain tissue also implicated *ST3GAL3*^16^.

To identify genetically regulated genes associated with TS, we conducted a TWAS of the current largest TS cohort of 4819 cases and 9488 controls^5^. Brain-specific panels were derived from the CMC and GTEx 53 v7. The TWAS revealed the expression of *FLT3* to be increased across many brain tissues in TS, with the largest effect in the dorsolateral prefrontal cortex (DLPFC). Given that *FLT3* is expressed in lymphoblasts, we additionally measured the RNA expression of *FLT3* in 100 lymphoblastoid cell lines (LCL;50 cases and 50 controls). Brain samples for TS are often difficult to acquire and not readily available in large sample sizes, which make LCL a useful source of information. Previous studies have demonstrated that ~23% of post-mortem brain tissue and lymphoblastoid cell lines have similar expression levels^17^. There was an increased expression in *FLT3* in LCL derived from TS cases, consistent with TWAS results. In conclusion, increased expression of *FLT3* was implicated through TWAS across several brain tissues and expression in LCL.

## Results

### Transcriptome-wide significant hits

To identify genes associated with TS, a TWAS was conducted using FUSION. The strongest significant hit was *FLT3*, with increased expression (*Z* = 4.67, *P* = 2.98E−06) in the DLPFC (Table 1). Interestingly, the gene also had increased expression in the brain cortex, hippocampus, anterior cingulate cortex, frontal cortex, cerebellum, and cerebellar hemispheres suggesting a global dysregulation across brain tissue types. The gene *DHRS11* was also implicated (*Z* = 4.26, *P* = 2.01E−05), although not genome-wide significant. An omnibus test using the GTEx and CMC brain tissue panels also identified the top two genes: *ATP6V0A2* (*P* = 3.70E−05) and *NEB* (*P* = 1.72E−04) (Table 1). Given the lower number of FDR-significant hits, we used a nominally significant threshold (FDR-corrected *p* value < 0.10) to assess pathways. We found that significant pathways were related to *FLT3* signalling, myosin binding, and microfilament activity (Supplementary Data 1).

**Table 1:** TWAS genes with association to Tourette’s syndrome.

Gene	Method	Tissue	*P* value	Permutation *P* value	*Z*-score
*FLT3*	Expression	Dorsolateral prefrontal cortex	3.04E−06	0.00616	4.6683
*FLT3*	Expression	Cortex	3.24E−06	0.01289	4.6551
*FLT3*	Expression	Hippocampus	7.87E−06	0.0108	4.4688
*FLT3*	Expression	Anterior cingulate cortex BA24	8.12E−06	0.007	4.462
*FLT3*	Expression	Frontal cortex BA9	8.30E−06	0.0185	4.4574
*FLT3*	Expression	Cerebellum	1.44E−05	0.011	4.3382
*DHRS11*	Expression	Substantia nigra	2.01E−05	0.0153	4.26398
*FLT3*	Expression	Cerebellar hemisphere	2.43E−05	0.0096	4.22163
*ATP6V0A2*	Omnibus	–	3.70E−05 (Nominally significant)	–	–
*NEB*	Omnibus	–	0.000172 (Nominally significant)	–	–
*MPHOSPH9*	Splicing	Dorsolateral prefrontal cortex	1.58E−05	0.01509	−4.317536
*FIP1L1*	Splicing	Dorsolateral prefrontal cortex	2.55E−05	0.00184	4.21065
*CSGALNACT2*	Splicing	Dorsolateral prefrontal cortex	3.39E−05	0.00090	4.14582

### Splicing in Tourette’s syndrome

Given the importance of alternative splicing in neurodevelopment and brain gene expression, a splicing TWAS was done to identify splicing events associated with TS. There were several significant genes after permutation. The top three hits were *MPHOSPH9* (*Z* = −4.32, *P* = 1.58E−05), *FIP1L1* (*Z* = 4.21, *P* = 2.55E−05) and *CSGALNACT2* (*Z* = 4.14, *P* = 3.39E−05) (Table 1). However, we also caution on the interpretability of the effect direction given that alternatively spliced exons are typically negatively correlated^18^.

### Fine-mapping of *FLT3* locus

**Fig. 1:**
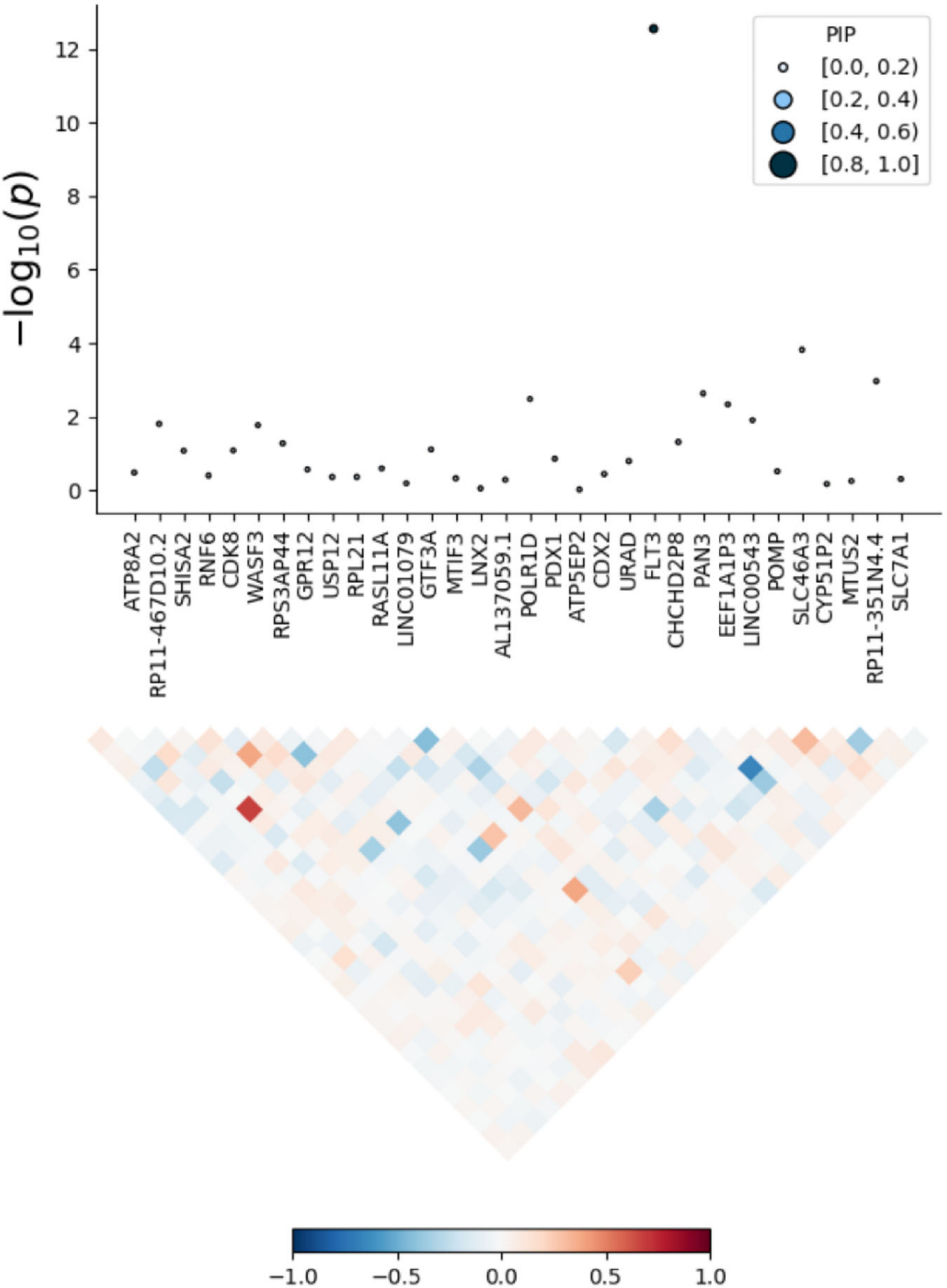
Fine mapping of chromosome 13 TWAS signal. PIP is the posterior inclusion probability. TWAS *p* values derived from FOCUS are on the *Y*-axis and genes within the locus are on the *X*-axis. The local correlation structure is shown in the bottom half of the figure. The PIP for *FLT3* was the highest.

To determine whether *FLT3* is the putatively causal gene on the DLPFC, FOCUS was used to assign a probabilistic inclusion probability for genes at the TWAS region^19^. For the region 13:27284583-13:29257379 (hg19 coordinates), the *FLT3* gene had the highest posterior inclusion probability (PIP) of 0.849 and was included in the 90% credible gene set (Fig. 1). The PIP is an inclusion probability (0–1), where a higher PIP may suggest higher chance of being potentially causal.

### RT-qPCR of *FLT3*

**Fig. 2:**
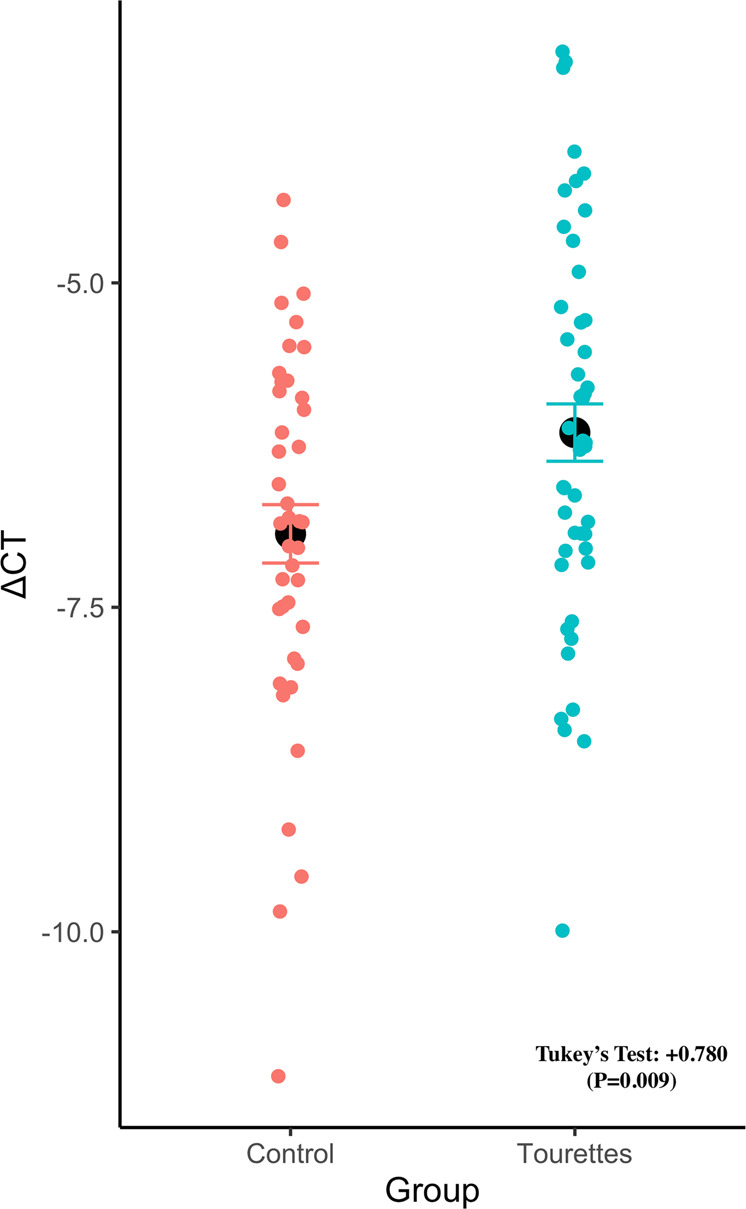
*FLT3* RNA Expression differences between Tourette’s Syndrome patients and controls in lymphoblastoid cell lines (LCL). TS LCL had higher expression of *FLT3* compared to controls after adjusting for plate, sex, and age. The black dot represents the mean of the data and error bars are ±SE.

Given that brain tissue for TS is difficult to acquire in large sample sizes, LCL were from TS patients and controls were used to assess the expression of *FLT3*. To test normality of qPCR data, a Shapiro–Wilk test was done. It was found that the ∆CT values (a measure of expression based on the difference in the number of PCR cycles required for the fluorescent signal to exceed background level) were normally distributed (*W* = 0.99, *P* = 0.70). Next, an ANOVA was done and determined that the disease status was statistically different in the dataset (*F* = 7.06, *P* = 0.0095). A Tukey’s test showed that TS patients had significantly higher expression of *FLT3* compared to controls, with a ∆CT difference 0.780 (*P* = 0.009) (Fig. 2). The corresponding fold change is +1.72 higher in TS than controls. The effect size was determined to be moderate-large (Cohen’s F, 0.30).

### Phenome-wide association study of *FLT3*

**Fig. 3:**
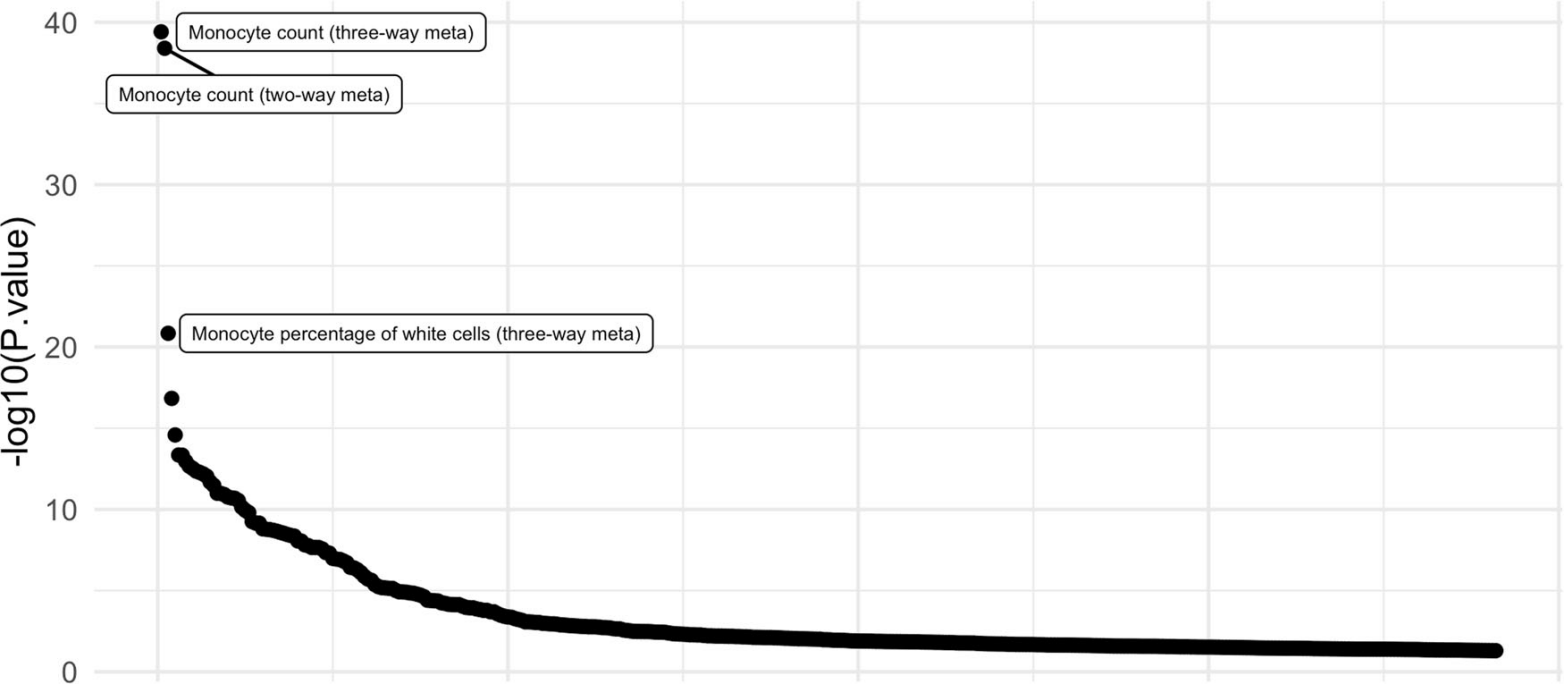
Regional phenome-wide association study (pheWAS) of *FLT3* for 2977 unique traits. Each point represents a trait. Only traits with an association (*P* < 0.05) were included for the plot.

To identify phenotypes associated with the top gene, a regional phenome-wide association study spanning the entire gene (pheWAS) was done for *FLT3*. The pheWAS identified several immunological traits associated with *FLT3* such as monocyte count (3.87E−40) and percentage of white blood cells (1.42E−21) (Fig. 3).

## Discussion

While recent GWAS for TS have successfully identified risk loci, the biological relevance of these associations remains unknown. Here, we conduct a TWAS using the summary statistics of over 14,000 individuals from the most recent TS GWAS^5^. This approach allows for imputation of expression by leveraging genotype-expression reference panels. From this, we identified increased *FLT3* expression as the top hit in the DLPFC and additionally found an increase in expression across most brain tissue types, suggesting global dysregulation. The global dysregulation may suggest that commonly TS-implicated brain areas such as the supplementary motor area could potentially have *FLT3* dysregulation^20^. Validation of expression in LCL prepared from TS cases found an increased or an increase in RNA expression compared to LCL from control individuals. The *FLT3* gene encodes for a tyrosine-protein kinase and it has been associated with inflammation and immune function^21,22^. This could point toward immunity in TS as a putative biological mechanism. The *FLT3* gene has been shown to be related to the developmental process of mouse brain, and its expression was markedly increased with age. Furthermore, the pheWAS identified that *FLT3* is associated with immunological traits such as monocyte count and white blood cell counts. However, we emphasize that the pheWAS pertains only to the top hit *FLT3* and not the rest of the genomic loci underlying TS risk. Interestingly, previous studies have demonstrated that TS patients have significantly higher levels of monocytes compared to healthy controls^23^. This could suggest dysregulation of monocytes partially due to increased expression of *FLT3*, which may contribute toward pathogenicity of TS. Alternatively, *FLT3* could also alter neuroimmune interactions, but further functional data would be needed to investigate this. Furthermore, fine-mapping the TWAS hit demonstrated that *FLT3* was in the 90% causal credible-set with a PIP of 0.849 for the DLPFC. This further stipulates that *FLT3* is the strongest putative gene at this locus. A previous study investigating transcriptomic differences of the basal ganglia between TS and controls found an enrichment of differentially expressed immune-related genes, reinforcing the potential importance of immune-related genes in TS^24^.

The splicing TWAS identified several putatively associated genes (*MPHOSPH9*, *FIP1L1*, and *CSGALNACT2*) associated with TS, suggesting that both splicing, and genetically regulated genes are potentially implicated in TS. The *MPHOSPH9* gene encodes for a protein that regulates cell cycling^25^. This gene has been implicated in multiple sclerosis, which is an inflammatory disease of the central nervous system^25^. In addition, *FIP1L1* is associated with pre-mRNA 3′-end formation and has been implicated in immunological function by cooperating with IL-5^26,27^. These findings could potentially support the hypothesis that the pathophysiology of TS may include or involve the immune system. Understanding the role of immunity in TS may elucidate the link between streptococcal infections and tic exacerbations as proposed in the pediatric autoimmune neuropsychiatric disorders associated with streptococcal infections (PANDAS) hypothesis^1,28^. It is also possible that these dysregulated may point to synaptic pruning and potentially overactive microglia instead of an immune response, which has been implicated in schizophrenia^29^. A previous study has also found that microglia-mediated neuroinflammation was found higher in the TS group in the bilateral caudate and bilateral lentiform nucleus and bilateral caudate nuclei compared to controls^30^. The *CSGALNACT2* gene encodes for chondroitin sulfate protein, which is involved in the brain extracellular matrix^31^. A previous meta-analysis of ADHD and TS showed implication of sulfuration of chondroitin, suggesting potential relevance^32^. These results build upon the GWAS study by suggesting that *FLT3* expression may be overexpressed in TS and identifying putative splicing gene targets to further investigate. Given the high degree of splicing in brain tissue, this area may prove fruitful for identifying novel gene targets^9^.

We conclude this study with some strengths, caveats and potential future directions. Strengths of TWAS include trying to unravel the biological relevance behind GWAS signals and identify gene targets for functional follow-up. Here, we were able to investigate the directional effects of *FLT3* and prioritize the gene over others within the same GWAS locus. For limitations, TWAS signals can putatively be confounded due to expression imputation from weighted linear combinations of SNPs. Because of this, some of these SNPs may be associated with non-regulatory mechanisms that inflate the test statistic. A second caveat is that there is currently no available replication cohort, given that the largest GWAS for TS was used for this study. Future work could look at integrating single-cell sequencing data with TS GWAS to determine single-cell cis-eQTL regulated genes. Furthermore, individual TWAS risk could be investigated in independent cohorts. A third caveat is that a given gene may be influenced by genetic regulators independent of cis-eQTLs and sQTLs but still have downstream effect on TS. Finally, the use of GWASAtlas for the pheWAS can present potential selection bias on which traits get included, however, it was used since it can be readily used by any investigators as more phenotypes get added. In conclusion, we identify the *FLT3* gene as likely involved in TS with increased expression found by TWAS and in lymphoblastoid cell lines of patients. We further identify several significant genes associated with aberrant splicing and point toward immunity in the pathogenesis of TS.

## Methods

### Genotyping data

Public summary statistics were obtained from the Psychiatric Genetics Consortium through the OCD & Tourette Syndrome group. Briefly, the summary statistics consists of a case-control GWAS for TS. Population stratification was accounted for through multidimensional scaling, and European individuals were retained. Imputation was done using the 1000 Genomes phase 1 haplotypes. Meta-analysis of different cohorts for the GWAS was done using an inverse variance model. Further details on the participant ascertainment and quality control steps are previously described in the 2019 TS GWAS^5^. The summary statistics were munged using LDSC and were used as the input for the subsequent transcriptomic imputation^33^.

### Transcriptomic imputation

Imputation was done by using reference panels from FUSION that were derived from consortia datasets of tissue-specific gene expression integrated with genotypic data. The CommonMind Consortium (CMC) and brain tissue panels from GTEx 53 v7 were used for a total of 14 tissue types and panels. To account for multiple hypothesis testing, *P* values were adjusted for false-discovery rate (FDR). FUSION was used to conduct the transcriptome-wide association testing and features that capture a significant heritability were used. The 1000 Genomes v3 LD panel was used for the TWAS. FUSION utilizes several penalized linear models, such as GBLUP, LASSO, Elastic Net. In addition, a Bayesian sparse linear mixed model is used. FUSION computes an out-sample *R*^2^ to determine the best model by performing a fivefold cross-validating of every model. After, a multiple degree-of-freedom omnibus test was done to test for effect in multiple reference panels. The threshold for the omnibus test was *P*  =  4.64E−06 (0.05/10,323 [number of genes tested]). Next, we sought to assess splicing events associated with TS. Splicing analysis was done using the CMC splicing imputation panel obtained from FUSION, following the same methods as described earlier, and an FDR *p* value < 0.05 was considered significant.

### Fine-mapping of TWAS associations

To address the issue of co-regulation and LD, we used FOCUS (Fine-mapping of causal gene sets) to model predicted expression correlations and to assign a posterior probability for causality in relevant tissue types^19^. Briefly, FOCUS prioritizes genes for each TWAS hit to be included in a 90%-credible set while accounting for pleiotropic SNP effects. The identical TWAS reference panels for FUSION were used as in the analysis described above.

### Phenome-wide association studies

To identify phenotypes associated with *FLT3*, a phenome-wide association study (pheWAS) was done. PheWAS was done using public data provided by GWASAtlas (https://atlas.ctglab.nl)^34^. Briefly, the pheWAS seeks to identify traits that are associated with *FLT3* by querying many different GWAS traits. This method seeks to prioritize phenotypes that may be associated with *FLT3*. Only publicly available GWAS summary statistics were included in the GWASAtlas database. Any GWAS based on immune-chip sequencing, whole-exome sequencing or replicable cohorts were excluded. At the time, there were 2977 unique traits and the Bonferroni-corrected threshold was set at 1.68E−5 (0.05/2977).

### Lymphoblastoid cell lines

Tourette’s patients were recruited at the TS clinic at the Montreal General Hospital and theAllen Memorial Institute. The family members were interviewed by a group consisting of a psychiatrist, neuropsychologist, and neurologist. Symptoms were evaluated with semi-structured interviews using standardized scales. All Tourette’s patients had definite TS based on the DSM-IV and the TS Classification Study Group. Severity of tics was evaluated using the Yale Global Tic Severity Scale. Patients were excluded if there was evidence of another neurological disorder that could mimic TS, or if they had neuroleptic-induced tardive dyskinesia, or tics secondary to head injury or drug abuse. Lymphoblastoid cell lines were prepared from consenting individuals. The study was approved by the institutional review board of McGill University (IRB00010120) and informed consent was obtained from patients. A total of 100 LCL (50 controls and 50 cases) were randomly selected. Cells derived from TS patients and controls were grown at 37 °C and cells were cultured for ~1 week prior to RNA extraction. Control LCL were selected if there is no clinical reporting of TS or any other psychiatric disorder. Similar ages and sex were chosen to match the TS samples.

### RNA extraction

RNA was extracted from the cells using the Qiagen RNAeasy Mini Kit. The RNA was subsequently stored in −80 °C after elution with RNAse-free water. One microgram of each sample of RNA was converted into cDNA using the SuperScript VILO cDNA Synthesis Kit by Thermo Fisher Scientific.

### Reverse-transcriptase quantitative qPCR

The cDNA was used to perform a Taqman qPCR using QuantStudio 7. The *FLT3* probe (Accession number: Hs00174690_m1) was used, and *POLR2A* (polymerase [RNA] II [DNA-directed] polypeptide) (Accession number: Hs00172187_m1) was used as the endogenous control. The thermocyler parameters consisted of the following: 2 min at 95 °C, and 40 cycles of 95 °C for 1 s and 60 °C for 20 s. The qPCR was performed in triplicate. The cycling parameters were A Shapiro–Wilk Test was done to determine the normality of the mean CT values data. Mean CT values were derived from averaging the triplicate CT values. An ANOVA was done using the model mean CT values ~ sex + plate + disease status + disease status:sex). Mean CT values were derived from averaging the triplicate CT values. Cohen’s F was used to determine the effect size of the data.

### Reporting summary

Further information on research design is available in the Nature Research Reporting Summary linked to this article.

## Supplementary information

Description of Additional Supplementary Files

Supplementary Data 1

Supplementary Data 2

Supplementary Data 3

Supplementary Data 4

Reporting Summary

## Data Availability

All other data are contained within the article or its supplementary data (Supplementary Data 2 and 3) and available upon reasonable request. Supplementary Data 2 and 3 contain the TWAS summary statistics from Table 1. Source data to generate Figs. 1–3 can be found in Supplementary Data 4. The TS GWAS summary statistics can be accessed freely from the Psychiatric Genomics Consortium at https://www.med.unc.edu/pgc/download-results/ (data 10.6084/m9.figshare.14672232)^5^.

## Code availability

Code is available from the corresponding author upon reasonable request.
