# Supplementary material for: Transcriptome-wide association study reveals increased neuronal FLT3 expression is associated with Tourette’s syndrome
Source: Commun Biol. 2022 Mar 30;5:289. doi: 10.1038/s42003-022-03231-0 (PMC8967882; doi:10.1038/s42003-022-03231-0)
Supplement: Supplementary file 1 — Description of Additional Supplementary Files [file 42003_2022_3231_MOESM1_ESM.pdf]

Description of Additional Supplementary Files

File name: Supplementary Data 1
Description: Significant pathways enriched amongst TWAS genes.

File name: Supplementary Data 2
Description: Summary statistics of TS TWAS

File name: Supplementary Data 3.
Description: Summary statistics of TS splicing TWAS

File name: Supplementary Data 4
Description: Source data for figures.
